# Modelling monthly influenza cases in Malaysia

**DOI:** 10.1371/journal.pone.0254137

**Published:** 2021-07-21

**Authors:** Muhammad Adam Norrulashikin, Fadhilah Yusof, Nur Hanani Mohd Hanafiah, Siti Mariam Norrulashikin

**Affiliations:** 1 Department of Mathematical Science, Universiti Teknologi Malaysia, Skudai, Malaysia; 2 Hospital Mersing, Mersing, Johor, Malaysia; Vellore Institute of Technology: VIT University, INDIA

## Abstract

The increasing trend in the number new cases of influenza every year as reported by WHO is concerning, especially in Malaysia. To date, there is no local research under healthcare sector that implements the time series forecasting methods to predict future disease outbreak in Malaysia, specifically influenza. Addressing the problem could increase awareness of the disease and could help healthcare workers to be more prepared in preventing the widespread of the disease. This paper intends to perform a hybrid ARIMA-SVR approach in forecasting monthly influenza cases in Malaysia. Autoregressive Integrated Moving Average (ARIMA) model (using Box-Jenkins method) and Support Vector Regression (SVR) model were used to capture the linear and nonlinear components in the monthly influenza cases, respectively. It was forecasted that the performance of the hybrid model would improve. The data from World Health Organization (WHO) websites consisting of weekly Influenza Serology A cases in Malaysia from the year 2006 until 2019 have been used for this study. The data were recategorized into monthly data. The findings of the study showed that the monthly influenza cases could be efficiently forecasted using three comparator models as all models outperformed the benchmark model (Naïve model). However, SVR with linear kernel produced the lowest values of RMSE and MAE for the test dataset suggesting the best performance out of the other comparators. This suggested that SVR has the potential to produce more consistent results in forecasting future values when compared with ARIMA and the ARIMA-SVR hybrid model.

## Introduction

Generally, influenza is categorised as an acute contagious viral respiratory disease that are usually characterized by the presence of fever, cough, sore throat, headache, and myalgia. Influenza viruses belong in the Orthomyxoviridae family and are further sub-divided into influenza A, B and C whereby the latter only causes mild sporadic disease. Each year, according to the World Health Organization (WHO), there are 5–10% and 20–30% new cases of influenza infection among adults and children, respectively [1]. This results in 3 to 5 million serious illnesses worldwide causing approximately 290,000–650,000 deaths [2]. Influenza viruses cause epidemics and pandemics and can escalate every year. This can result in high rate of hospitalisation among susceptible community, which in turn places economic burdens on families and society [3].

The effects of influenza epidemics and pandemics can be more serious in developing countries due to resource scarcity and deprivation in health and nutrition funding. Therefore, WHO highly recommends for more research to be produced for the developing countries on influenza epidemiology and disease incidence [4]. In temperate regions, seasonal influenza typically occurs every year in the late fall or winter but in tropical and subtropical regions, the seasonality of influenza cannot be clearly distinguished. The research data in Malaysia suggests that influenza can occur year-round, though records of seasonal peak for presence or absence are uncertain. This trend is almost similar with other tropical countries [5–7].

Current literature lacks the implementation of time series forecasting towards local Malaysian data. Time series forecasting uses a forecast model to estimate the values of a continuous variable which is also called as a response variable or output variable, based on historical data [8, 9]. Exponential smoothing, Autoregressive-Integrated-Moving Average (ARIMA), and Autoregressive Conditional Heteroscedastic (ARCH) are some of the widely used approaches in univariate time series models [10]. Through the method employed by Zhang (2003), this paper intends to perform a hybrid ARIMA-SVR approach in forecasting monthly influenza cases in Malaysia. ARIMA model (using Box-Jenkins method) and Support Vector Regression (SVR) model were used to capture the linear and nonlinear components of the monthly influenza cases, respectively. Hence, the forecast performance of the hybrid model was expected to be improved.

Through this paper, we hope that the forecasting model can aid in predicting influenza outbreak in Malaysia. This will help in resource mitigation since the influenza outbreak can cause sudden increase in demand for drugs like Oseltamivir. Upcoming influenza outbreak forecasting is one of the most challenging public health concerns and predicting future outbreak plays a vital role in planning and managing healthcare expenditure. In addition, accurate recognition of influenza outbreaks is important for public health authorities to effectively implement strategies to control outbreaks and to help reduce the effects of diseases through preventive measures. Hence, a reliable detection system for timely detection of influenza outbreaks should be provided by identifying the main tools for detecting outbreaks in public health surveillance systems using real data testing. This paper is arranged as into a few sections. Section 2 discusses the methodology used in this study while Section 3 describes the experimental settings which include the data set, forecast model, procedures, and performance evaluation. The results, discussion and lastly the conclusion of this research are presented in Section 4 and 5.

This study attempted to recreate the “linear and nonlinear” time series forecasting method in disease prediction by employing the method of ARIMA and SVR. The advantage of using both ARIMA and SVR is that they can be utilized together in creating a better predictive model. Although many studies have shown the effectiveness of using this hybrid method in forecasting, they are seldomly used predicting disease outbreak in a local setting. Hence this study aimed to bridge this gap of knowledge. The results of this forecasting would provide an input on resource management and stock keeping not only for hospitals in general, but also for the distributors of medications related to influenza management.

## Methodology

Four forecasting methods were employed namely the Naiive, ARIMA, SVR and hybrid ARIMA-SVR. In this study, the most common ARIMA approach was used. Monthly influenza cases that have been transformed using min-max normalization were used as the input to construct these models. Descriptions of the implemented methods including their application in this study are discussed in the following sections, and the function of each method used are summarized in Table 1.

**Table 1:** Summary of the functions of the models used in this experiment.

Model	Function
**Naiive**	Baseline model for comparison of the models develop. Advanced and complicated models developed must outperform Naiive model.
**ARIMA**	Will capture the linear component of the influenza outbreak in Malaysia.
**SVR**	Will capture the non-linear component of the influenza outbreak in Malaysia.
**ARIMA-SVR Hybrid**	Combination of the linear and non-linear component of the model. ARIMA will be used as the initial model and residuals will be modelled using SVR.

### Naiive model

In this study, the Naiive model was used as a benchmark model to calculate measurement error [11]. Naiive model suggests that the future values will be the same as the past values. Hence, monthly influenza cases in the month of January for this research was expected to be the same as in February, and the number of cases in February would be the same as March. Having a simple benchmark model provides an indication that that any models that are more sophisticated should outperform this strategy. The Naiive method is recommended by some researchers as a measure of forecasting difficulty between series since a more volatile series forecasting would yield worse performance measure as compared to a more stable series.

### ARIMA model

To construct an ARIMA (p, d, q) model, parameters of p, d, and q must be identified beforehand. The parameters of p, d, and q refer to autoregression, differencing and moving average (MA) process of the model respectively [12]. ARIMA model can be generally defined as follows:

$$\Psi (B){\left(1-b\right)}^{d}y_{t}=\theta (B)\varepsilon_{t}$$ (1)

where *y*_*t*_ and *ε*_*t*_ represent the actual values of monthly influenza cases with random error terms on day *t*. B is defined by *By*_*t*_ = *y*_*t*−1_. Ψ(*B*) and *θ*(*B*) are defined as follows:

$$\Psi (B)=1-\varphi_{1}B-\varphi_{2}B^{2}-\varphi_{3}B^{3}-\cdots -\varphi_{p}B^{p}$$ (2)

$$\Psi (B)=1-\theta_{1}B-\theta_{2}B^{2}-\theta_{3}B^{3}-\cdots -\theta_{q}B^{q}$$ (3)

The implementation of an ARIMA model involves a four-step cycle:

1. Stationarity of the monthly influenza cases. Augmented Dickey-Fuller (ADF) test is performed to examine whether the time series is stationary or otherwise. If the series is not stationary, differencing is applied to the series until it is stationary.
2. Structure and parameter estimation for the ARIMA model. d is referred to as the differencing order derived in Step 1. Autocorrelation Function (ACF) and Partial Autocorrelation Function (PACF) plots are used to identify parameters p and q. Least square estimation is used to further estimate the parameters according to Bayesian Information Criterion (BIC).
3. The ARIMA models developed are validated and tested on the residual of the series. If the residual term passes the white noise test, then the ARIMA models developed can be assumed to be adequate.
4. Future data is forecasted.

### Support Vector Regression (SVR) model

Originally proposed by Vapnik (1995), SVR is based on the structured risk minimization which minimizes the upper bounds of generalization error using a subset of data points known as the support vector [13]. The assumption is that training dataset has pairs of (*x*_1_, *y*_1_), (*x*_2_, *y*_2_) …, and (*x*_*n*_, *y*_*n*_), where *x*_*i*_ ∈ *R*^*n*^ and *y*_*i*_ ∈ *R*^*n*^ are the input vectors and related output values respectively. By mapping *x*_*i*_ into a high dimensional feature by a non-linear function *φ*(*x*), SVR function can be expressed as:

$$f(x)=w^{T}\varphi (x)+b$$ (4)

where *f(x)* denotes the prediction, *φ*(*x*) is the feature function, while *w* and *b* are the corresponding coefficients [13]. SVR can be further expressed into the following equation for the parameters that can be used to optimize the model:

$$f(x)=\sum_{i=1}^{n}(\alpha_{i}-\alpha_{i}^{*})k(x_{i},x_{j})+b$$ (5)

where *α*_*i*_ and $\alpha_{i}^{*}$ are the lagrange multiplies; *k*(*x*_*i*_, *x*_*j*_) which is a kernel function. In this study, a linear kernel function was used. Linear kernel function is advantageous over the other kernel functions due to its simplicity in variables optimisation and it is also faster to compute.

### Hybrid ARIMA-SVR approach

**Fig 1:**
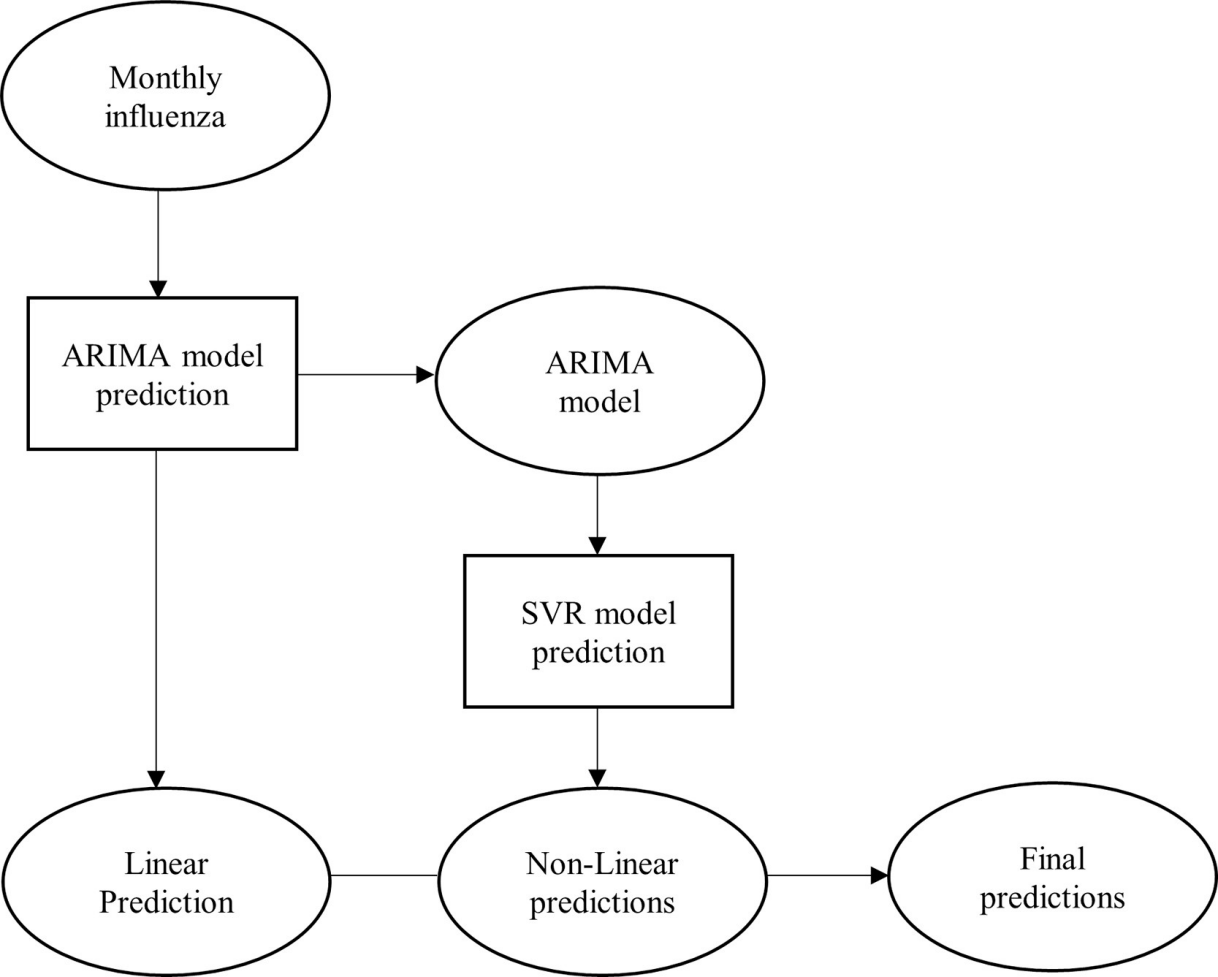
Flow chart for the development of ARIMA-SVR hybrid forecasting model.

In this hybrid model, both ARIMA and SVR models were used to model the linear and non-linear components in the monthly influenza cases. The flow for the development of the hybrid model is illustrated in Fig 1. The hybrid model can be expressed as:

$$y_{t}=L_{t}+N_{t}$$ (6)

where *y*_*t*_ is the original value on month *t*; *L*_*t*_ and *N*_*t*_ are the linear and non-linear components respectively. Residuals, *r*_*t*_ are obtained after modelling the linear model using the following equation:

$$r_{t}=y_{t}+{\hat{L}}_{t}$$ (7)

The value for the residuals *r*_*t*_ is then be estimated using SVR modelling process. ${\hat{L}}_{t}$ and ${\hat{r}}_{t}$ are the forecasted result from *L*_*t*_ and *r*_*t*_ respectively. *L*_*t*_ is forecasted using ARIMA while *r*_*t*_ uses SVR. Hence, the final model to predict the monthly influenza cases can be defined as the following:

$${\hat{y}}_{t}={\hat{L}}_{t}+{\hat{r}}_{t}$$ (8)

### Experimental settings

#### Data set and pre-processing

**Fig 2:**
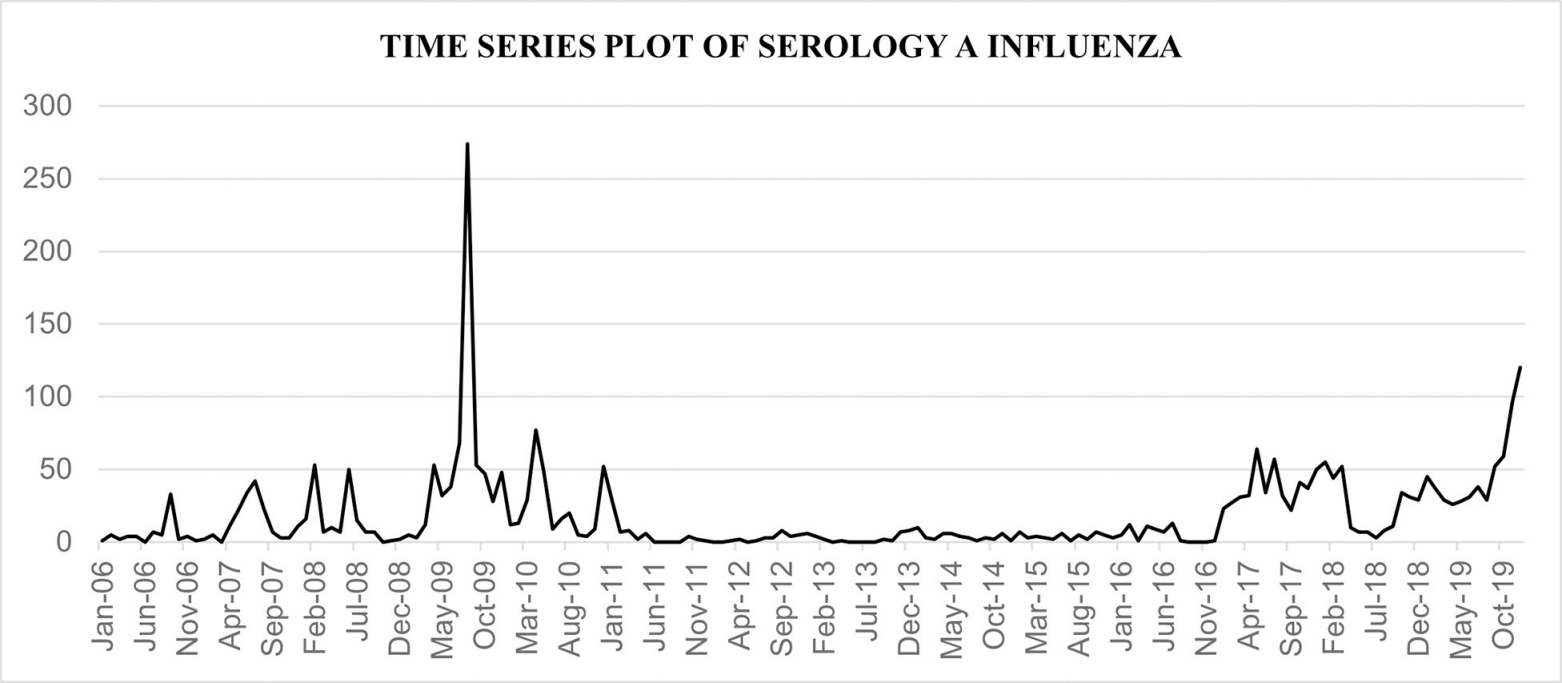
A time series plot for the number of influenza cases in Malaysia from January 2006 until December 2019.

The data of the influenza cases in Malaysia from the year 2006 until 2019 were obtained from World Health Organization (WHO) website which is publicly accessible using the following link https://apps.who.int/flumart/Default?ReportNo=12. Weekly Influenza serology A data was used and recategorized into monthly observations using pivot table in Microsoft excel. The time series plot for the whole dataset is illustrated in Fig 2 below. Table 2 shows the statistical descriptions of the data. It can be observed that there was a large difference between the minimum and maximum values of the monthly influenza cases in Malaysia.

**Table 2:** Statistical description of data set.

Sample size	Minimum	Maximum	Median	mean	Standard deviation	Skewness	Kurtosis
168	0	274	7.00	17.64	28.58655	4.903041	37.28069

The datasets were split into training and testing dataset for validation purpose. The training datasets were taken from the period of January 2006 until December 2016 while testing dataset utilised the data from the period of January 2017 until December 2019. Previous literature suggested the use of scaling methods to improve on prediction accuracy and enhancing forecast performance [14, 15]. In this study, the data were processed through min-max normalization as illustrated in Eq 9 where *y*_*min*_ and *y*_*max*_ are the minimum and maximum values for influenza cases according to the training dataset respectively. The residual series have also went through similar process as described in the equation below.


$${\tilde{y}}_{t}=\frac{y_{t}-y_{min}}{y_{max}-y_{min}}$$ (9)

#### Influenza model

Autoregressive (AR) models, Moving Average (MA) models, the AR and MA (ARMA) models combination, Autoregressive Integrated Moving Average (ARIMA) models or Seasonal Autoregressive Integrated Moving Average (SARIMA) models are evaluated according to the obtained dataset. Stationarity testing using ADF test is carried out to identify the stationarity of the series. If the data is found to be non-stationary, the first or second order differencing would be performed accordingly. The model order is identified using ACF (Autocorrelation Coefficient Function) and PACF (Partial Autocorrelation Function) plots. ACF and PACF of the transformed dataset of the time series are used to identify the order of p and q the models, respectively. To choose the best model, AIC (Akaike’s Information Criterion) value is usually used to evaluate model effectiveness and selection. Residual checking is performed for each of the model constructed using L-Jung Box test of the residuals. Once the model satisfies model assumptions, model efficiency will be calculated based on the fitted data and actual data using the train and test data.

With respect to influencing factors for the prediction of the future values, previous studies highlighted that lagged values can be used as predictor variables [16, 17]. Based on this, the developed SVR and hybrid model use the lagged values as an input variable. Consequently, for the SVR model, the assumption made is that the number of influenza cases on month *t* is a function of the visits in a past time windows (i.e, past *n* months) as expressed in Eq 10 as follows:

$${\tilde{y}}_{t}=f({\tilde{y}}_{t-1},{\tilde{y}}_{t-2},\;\ldots \;,{\tilde{y}}_{t-n})+\varepsilon_{t}$$ (10)

where ${\tilde{y}}_{t-1},{\tilde{y}}_{t-2},\;\ldots \;,{\tilde{y}}_{t-n}$ are the transformed numbers of inluenza cases on day *t-*1, *t-*2, *…*, *t-n* and *ε*_*t*_ represents the idiosyncratic demand shock. The forecasting model for the residual series are defined in the similar manner according to the residual series data set.

**Fig 3:**
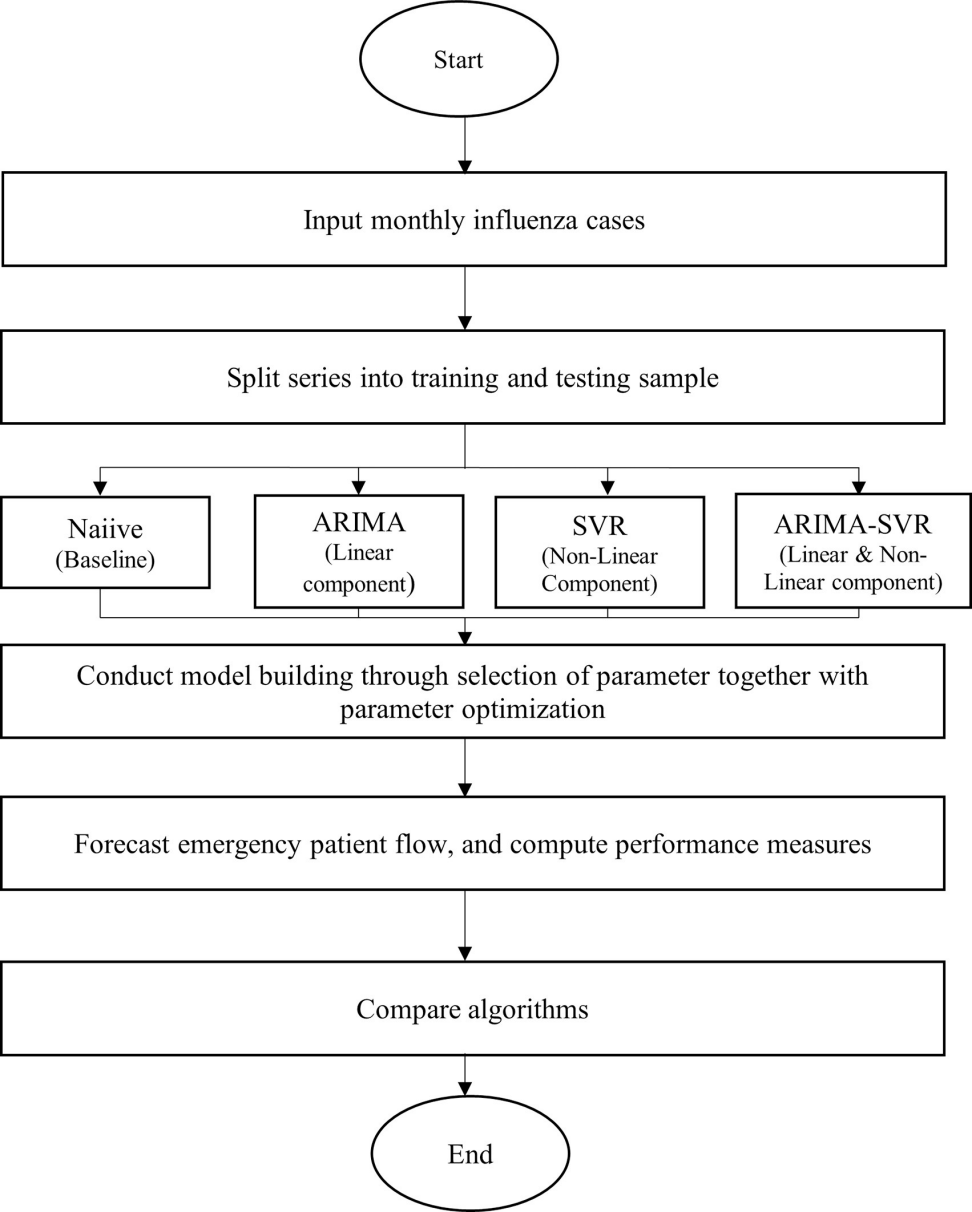
Procedure for development and comparing models in forecasting monthly influenza cases.

*Experimental procedure*. As discussed in the previous section, Naiive, ARIMA, SVR and hybrid ARIMA-SVR have been implemented in this study. All the models were developed using R software. Fig 3 shows the experimental procedure involved in this study. The experimental procedures involved are listed as follows:

1. Monthly influenza cases were split into training data set and testing data set. The first 132 observations (78.57% of the data) from January 2006 until December 2016 were used as the training data set while the remaining were used as the test data set for validation.
2. The parameters of the ARIMA model were determined using ACF and PACF plot. The best combinations of p, d, q parameters were determined through the evaluation of AIC. A baseline SVR (with cost and epsilon of 1.0 and 0.1 respectively) was used to compare every models that has been developed using hyperparameter optimization using 10-fold cross validation approach. Grid search was also performed to search for values of hyperparameters which mainly consisted of the cost and epsilon that gives the best output performance.
3. Models were then tested on their fitted values against the input values, residual checking for validation and the performance measures were computed.
4. Models were then re-evaluated and validated through forecasted or predicted values against testing data set.

*Performance measures*. Some of the commonly used forecast accuracy methods to measure model performance include Mean Absolute Percentage Error (MAPE), Root Mean Squared Error (RMSE) and Mean Absolute Error (MAE) as expressed in the series of equation below. These performance measures are widely used as the common measurement to observe the performance of the models. A model is said to be of a higher prediction accuracy when the value is close to zero (0).


$$MAPE=\frac{1}{N}\sum_{i=1}^{N}\left|\frac{y_{t}-y_{t}^{\prime }}{y_{t}}\right|\times 100\%$$ (11)

$$RMSE=\sqrt{\frac{\sum_{i=1}^{N}{\left(y_{t}-y_{t}^{\prime }\right)}^{2}}{N}}$$ (12)

$$MAE=\frac{\sum_{i=1}^{N}\left|y_{t}-y_{t}^{\prime }\right|}{N}$$ (13)

Where *y*_*t*_ is the actual data, $y_{t}^{\prime }$ is the fitted or forcasted data and *N* is the number of data points in the time series that will create the mean value for the error.

## Results and discussions

**Fig 4:**
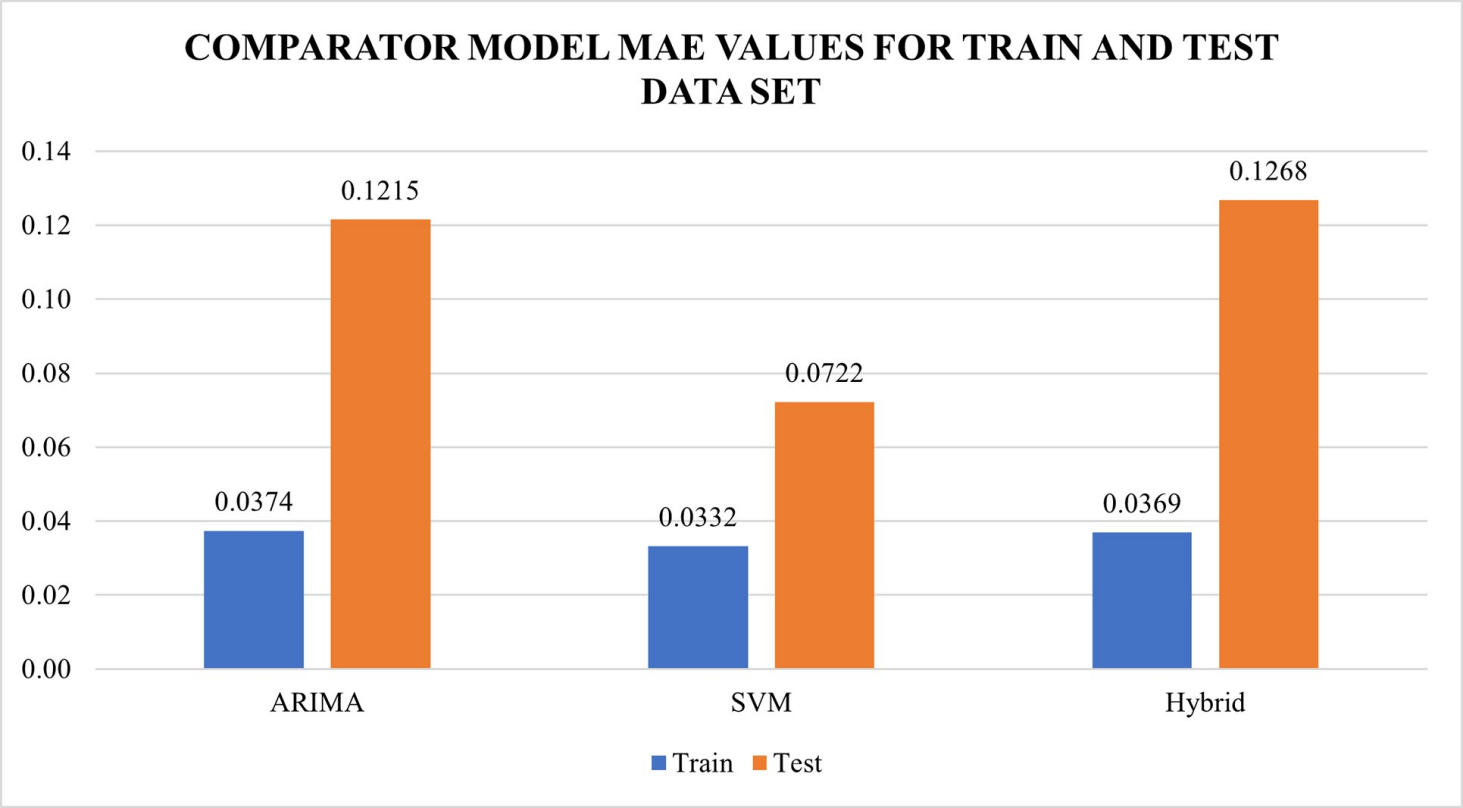
MAE values for both test and training dataset for ARIMA model, SVR model and ARIMA-SVR hybrid model.

**Fig 5:**
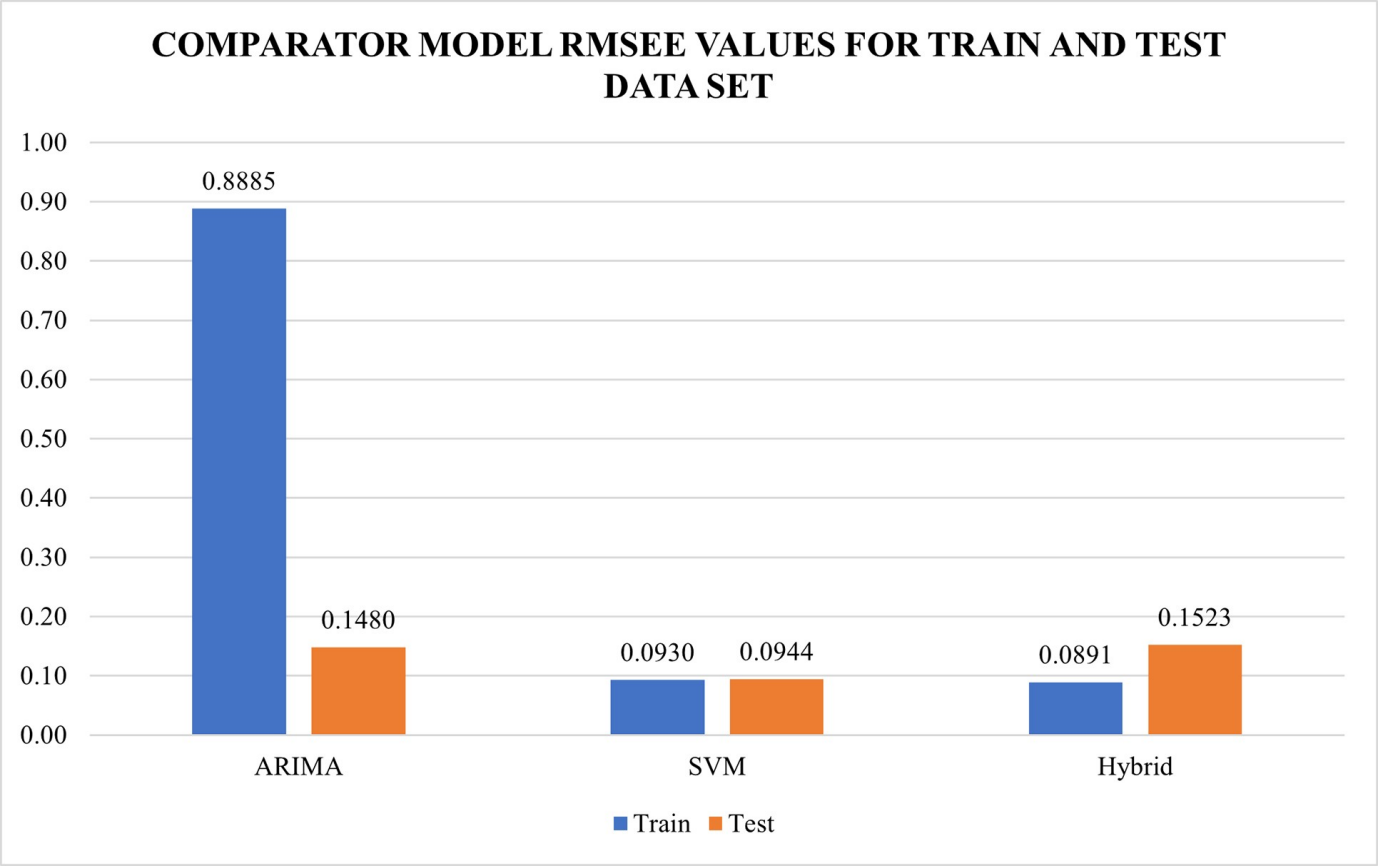
RMSE values for both test and training dataset for ARIMA model, SVR model and ARIMA-SVR hybrid model.

As discussed, the models have been compared against each other using performance measures namely RMSE and MAE. MAPE was not used as a performance measure since there were a few zero (0) values in the data set that caused the results of the MAPE to yield infinity. Validation using the residual values of the models for ARIMA, SVR and ARIMA-SVR developed was done. All of the residuals of the model was checked using autocorrelation function (ACF) and Q-Q plot. Residuals from the best models developed from ARIMA, SVR and ARIMA-SVR hybrid were found to be uncorrelated and normally distributed. The summary of performance measures are illustrated in Figs 4 and 5. Based on the observations, the monthly influenza cases have been successfully forecasted using the three comparator models as all the models have outperformed the benchmark model (Naiive model) as shown in Table 3. For the train dataset, RMSE and MAE of the comparator models namely the ARIMA, SVR and ARIMA-SVR hybrid ranged from 0.0891 to 0.8885 and 0.0332 to 0.0374 respectively. Meanwhile in the test dataset, RMSE and MAE values for comparator model ranged from 0.0722 to 0.1268 and 0.0944 to 0.1523 respectively. SVR with linear kernel produced the lowest values of RMSE and MAE for the test dataset suggesting the best performance out of the other comparators. This suggests that SVR produces a more consistent results in forecasting future values as compared to the ARIMA and the ARIMA-SVR hybrid model.

**Table 3:** Summary of MAE and RMSE values for train and test data set for the models developed as compared to benchmark model.

	Train		Test	
Model	MAE	RMSE	MAE	RMSE
**Naiive**	0.0409	0.0999	0.0409	0.0999
**ARIMA**	0.0374	0.8885	0.1215	0.1480
**SVM**	**0.0332**	0.0930	**0.0722**	**0.0944**
**Hybrid**	0.0369	**0.0891**	0.1268	0.1523

In a study by [18], it was suggested that the ARIMA-SVR hybrid model tends to produce better results in predicting daily emergency patient flow. In the model developed in their study, different kernel functions in both their basic SVR model and ARIMA-SVR model were used. Linear kernel was used in this study instead of the Radial Basis Function (RBF) Kernel as RBF might influence the forecast accuracy. The residual series is more optimized using a higher dimensional kernel such as the RBF Kernels to produce more accurate prediction of the residuals. Higher dimensional kernels might be better suited for the separation of the regression for the residuals of the ARIMA-SVR hybrid model. Besides optimizing the kernel function, the choice of model hyper-paramter optimisation should also be explored to yield a better model. Liu et. al. (2013) proposed an RGA-SVR method by employing genetic algorithm instead of the traditional 10-fold cross validation technique to tune the hyperparameter [19]. In their study, it was observed that the model performed better than the traditional SVR and back-propagation Neural Network (BP-NN).

The choice of predictor variables might also influence the accuracy of the result. In a research by Zhang, et. al. (2018), both lagged values and time characteristics that considered the seasonal components as well as holiday effects into their SVR model has been used [18]. Different lags could also be used to improve the prediction of the SVR and ARIMA-SVR model. In this study, a time lag of one value was used to predict future outcome. Further exploration of the correlation of different lags towards future values should be explored in the future to create a better predictor variable for the execution of SVR and ARIMA-SVR hybrid.

Influenza is normally observed in Malaysia year-round with no definite seasonal trends [20]. Influenza A is typically reported more often than influenza B but there can be significant year-to-year variability. A total of 2963 cases of influenza infection was reported in Malaysia from January 2006 until December 2019 with the minimum of 0 case and maximum of 274 cases per month. From these cases, the highest number of cases was reported in 2009 which summed up to 661 cases, or 22.3% from total cases. The 2009 H1N1 pandemic was the first global pandemic since 1968, and certainly raised public and health-care awareness on the severity of the disease. In terms of forecasting, it was almost universally accepted that the best particular strategy to describe every case could not be concluded [21]. This is mainly because a real-world problem is always dynamic in nature and various patterns cannot be captured equally by any single model. Hence, it is a process of trial and error where the researcher needs to find the best suitable and accurate model for forecasting.

## Conclusion

The rate of morbidity, mortality, and economic burden caused by influenza are very significant. Accurate disease occurrence timing and severity predictions will provide useful advance information to the public health community for them to prepare and implement the necessary measures. In subtropical and tropical areas, influenza epidemics can occur with varying epidemic severity throughout the year and this irregularity makes it more difficult to produce precise forecasts. For this study, the ARIMA-SVR hybrid model was proposed to be used for monthly influenza cases forecasting. Although it was assumed that ARIMA-SVR hybrid would outperform ARIMA and SVR model as it captured both linear and non-linear relationship of the data, SVR produced the best performance out of all the model executed. Higher dimensional kernel functions, different optimization strategy and different lags as predictor variable can be explored further to optimize the hybrid model developed. These findings are crucial in the application of resource planning and decision making, thus improving resource allocation to meet future demands. This is to increase the survival rate and assist healthcare agencies to prepare adequate resources to cater for future disease outbreaks. Future work should include other methods of machine learning for the hybrid models such as the implementation of Genetic Algorithm for model optimisation and the use of different lags for SVR model component and investigate its improvements from current methods.

## Supporting information

S1 Dataset Data for influenza serological A processed into months using pivot table. (CSV)
